# From Precocious Puberty to Infertility: Metabolic Control of the Reproductive Function

**DOI:** 10.3389/fendo.2013.00043

**Published:** 2013-04-02

**Authors:** Jennifer W. Hill, Meenakshi Alreja, Carol F. Elias

**Affiliations:** ^1^Department of Physiology and Pharmacology, University of ToledoToledo, OH, USA; ^2^Department of Obstetrics and Gynecology, University of ToledoToledo, OH, USA; ^3^Department of Psychiatry, Yale UniversityNew Haven, CT, USA; ^4^Department of Molecular and Integrative Physiology, University of MichiganAnn Arbor, MI, USA

Reproduction is calorically expensive. The energy demands of mate seeking, gamete production, pregnancy, and lactation require increased food consumption and appropriate regulation of energy expenditure. Therefore, control of the reproductive function by the brain must be responsive to the metabolic state of the animal. Conversely, when survival is threatened by insufficient fuels or increased energy demands, males and females of most species divert energy away from reproduction by reducing copulatory motivation and behavior, halting ovulation, terminating pregnancies, or ceasing lactation. In addition, when prepubertal animals, including humans, are exposed to energy deprivation, the onset of puberty is delayed or even blocked, until a favorable energy balance is achieved. These mechanisms serve to optimize reproductive success in environments where energy availability fluctuates. Recent studies suggest that excess body fat can trigger early onset of puberty, especially in females. In males, on the other hand, the prevalence of delayed puberty is about fivefold higher than in females, indicating sexual dimorphism in the sensitivity of the reproductive axis to metabolic cues. Understanding the interaction between energy balance and fertility has critical implications for the treatment reproductive deficits caused by metabolic dysfunction.

Given the involvement of the hypothalamus in the management of food intake, energy use, reproductive behavior, and the hormonal control of gametogenesis and ovulation, ongoing studies are focused on the interplay between the hypothalamic circuits driving these functions. Gonadotropin releasing hormone (GnRH) neurons are specialized neurons often described as the “master regulators” of the hypothalamus-pituitary-gonads (HPG) axis. The intermittent release of GnRH controls pituitary release of luteinizing hormone (LH) and follicle stimulating hormone (FSH) and, by extension, function of the gonads. Surprisingly, few GnRH neurons are sufficient to initiate puberty in males and females and to maintain fertility in the male. However, more are required for females to generate LH surges and ovulate. These additional GnRH neurons may modulate GnRH pulsatility in response to environmental, nutrition, stress, or other cues conveying adverse situations. However, GnRH neurons on their own seem to sense few metabolic cues. Instead, neighboring neurons and glia may perceive circulating factors, such as leptin, insulin, and ghrelin that serve as signals of the nutritional state of the individual. If these cells are not able to sense metabolic cues, for example in states of insulin or leptin resistance, the repercussions may include both imbalanced metabolic homeostasis and reproductive dysfunction. Indeed, leptin-deficient patients become hyperphagic, massively obese, and infertile. This eBook has assembled multidisciplinary specialists to provide up-to-date information on recent advances in understanding the complex physiologic interaction between metabolism and reproduction.

In the initial article, True et al. (2011) discuss the role of the adipocyte hormone leptin as a key metabolic signal and predominant focus of interest in the field. In their review, it is emphasized that although leptin may be an important permissive signal for reproductive function as indicated by many years of research, factors other than leptin must critically contribute to negative energy balance-induced reproductive inhibition. Schneider et al. (2012) call attention to the “metabolic hypothesis,” which predicts that sensory systems monitor the availability of oxidizable metabolic fuels and allow behavioral responses to optimize reproductive success. Following these provocative introductory articles, three reviews discuss the role of specific groups of neurons in this physiologic regulation. Bianco (2012) highlights the Kisspeptin system as the converging target of environmental, metabolic, and hormonal signals, and proposes a potential correlation between the existence of a sexual dimorphism of pubertal disorders in children of different ethnicities and the sexually dimorphic expression of kisspeptin neurons. Supported by recent genetic studies, Xu et al. (2012) focused their review on two sets of hypothalamic neurons: the pro-opiomelanocortin (POMC) neurons in the arcuate nucleus and the steroidogenic factor-1 (SF1) neurons in the ventromedial hypothalamic nucleus. Their discussion calls attention to exciting new findings showing that disruption of metabolic signals (e.g., leptin and insulin) or reproductive signals (e.g., estradiol) in these neurons leads to impaired regulation of both energy homeostasis and fertility. Donato and Elias (2011) discuss the role of the ventral premammillary nucleus as integrator of environmental, metabolic, and reproductive cues, and its emergence as a critical previously unrecognized hypothalamic site linking metabolism and reproduction. Acosta-Martínez (2012) proposes a role for phosphatidylinositol-3-kinase (PI3K) signaling pathway as potential integrator of a number of peripheral metabolic cues, including insulin and leptin, in the metabolic control of the reproductive function. Tolson and Chappell (2012) offer an insightful discussion on pubertal timing, outlining a potential role of endogenous timing mechanisms including cellular circadian clocks in pubertal initiation. They propose that these clocks may be altered by metabolic factors leading to reproductive deficits. In a provocative review, Clasadonte et al. (2011) discuss the action of non-neuronal components in GnRH regulation. They suggest that synaptically associated astrocytes and perijunctional tanycytes are integral modulatory elements of GnRH neuronal function at the cell soma/dendrite and terminal levels. Finally, two important articles call the attention to differences in the metabolic modulation of the reproductive physiology in different species. Klingerman et al. (2011) highlight the metabolic influence on sexual behavior, and food intake or food hoarding in hamsters, and suggest a role for neuropeptide Y (NPY) and gonadotropin inhibiting hormone (GnIH) expressing cells in these processes. Amstalden et al. (2011) emphasize observations made in ruminant species in a very welcome comparative perspective. Clearly, research examining the metabolic control of reproduction is advancing at a rapid pace. The articles in this eBook highlight some of the most critical and intriguing areas for future study.
